# Association of Hypothyroidism and the Risk of Cognitive Dysfunction: A Meta-Analysis

**DOI:** 10.3390/jcm11226726

**Published:** 2022-11-14

**Authors:** Yuanyuan Ye, Yiqing Wang, Shiwei Li, Jiyun Guo, Li Ding, Ming Liu

**Affiliations:** Department of Endocrinology and Metabolism, Tianjin Medical University General Hospital, 154 Anshan Road, Heping District, Tianjin 300052, China

**Keywords:** hypothyroidism, cognitive dysfunction, dementia, risk factor, meta-analysis

## Abstract

Objectives: The purpose of this meta-analysis was to assess whether there is an association between hypothyroidism and the risk of cognitive dysfunction. Methods: PubMed, Cochrane Library, and Embase were searched for relevant studies published from database inception to 4 May 2022, using medical subject headings (MeSHs) and keywords. Results: Eight studies involving 1,092,025 individuals were included, published between 2010 and 2021. The pooled analysis showed that there was no association between hypothyroidism and cognitive dysfunction (OR = 1.13, 95% CI = 0.84–1.51, *p* = 0.426), including both all-cause dementia (OR = 1.04, 95% CI = 0.76–1.43, *p* = 0.809) and cognitive impairment (OR = 1.50, 95% CI = 0.68–3.35, *p* = 0.318). Neither overt hypothyroidism (OR = 1.19, 95% CI = 0.70–2.02, *p* = 0.525) nor subclinical hypothyroidism (OR = 1.04, 95% CI = 0.73–1.48, *p* = 0.833) was associated with cognitive dysfunction. Neither prospective cohort (OR = 1.08, 95% CI = 0.77–1.51, *p* = 0.673) nor cross-sectional studies (OR = 1.23, 95% CI = 0.63–2.42, *p* = 0.545) had any effect on the association. Interestingly, the risk of cognitive dysfunction was significantly increased in the group not adjusted for vascular comorbidity (OR = 1.47, 95% CI = 1.07–2.01, *p* = 0.017), while it was reduced in the adjusted group (OR =0.82, 95% CI = 0.79–0.85, *p* < 0.001). Conclusions: This meta-analysis shows that hypothyroidism was associated with a reduced risk of cognitive dysfunction after adjustment for vascular-disease comorbidities. More prospective observational studies are needed in the future to investigate the relationship between hypothyroidism and cognitive dysfunction.

## 1. Introduction

Dementia is a clinical syndrome that is characterized by a series of symptoms and signs, leading to complex cognitive decline. It has become one of the biggest health threats in old age and has substantial socioeconomic costs [1]. It is estimated that the number of individuals who develop dementia will rise to 131.5 million by 2050 [2]. Mild cognitive impairment (MCI) is a type of cognitive decline that is abnormal for age but largely preserves functional activities; the progression rate from cognitive impairment to dementia in the general population older than 65 years is around 6–10% per year [3]. Thyroid dysfunction is considered a potentially reversible cause of cognitive decline. Therefore, the guidelines recommend thyroid-function screening tests as an important part of the diagnostic test for dementia [4]. Many studies have investigated whether hypothyroidism is associated with an increased risk of cognitive impairment. However, the results are conflicting. Overt hypothyroidism has previously been considered to lead to cognitive impairment, but a recent study [5] showed that no association between overt hypothyroidism and overall cognitive decline could be observed. Similarly, subclinical hypothyroidism is common in the general population, but whether subclinical hypothyroidism is associated with cognitive impairment remains controversial [6,7,8,9,10,11]. Some studies suggest that subclinical hypothyroidism is not associated with an elevated risk for dementia, but the latest comprehensive evidence is still needed to reach conclusions. Without strong evidence, current guidelines recommending screening for thyroid dysfunction in older adults with cognitive decline cannot be supported.

## 2. Methods

This study was conducted in accordance with the Preferred Reporting Items for Systematic Reviews and Meta-Analyses (PRISMA) guidelines [12], and the protocol was registered in the International Prospective Register of Systematic Reviews (PROSPERO) (CRD42022330577).

### 2.1. Search Strategy

We systematically searched the PubMed, Cochrane Library, and Embase, without language restrictions, from their inception to 4 May 2022. The Medical Subject Heading (MeSH) terms and keywords used in the search were as follows: (“hypothyroidism” OR “hypothyroidism*” OR “thyroid stimulating hormone deficienc*” OR “TSH deficienc*”) AND (“dementia” OR “Alzheimer Disease” OR “dementia*” OR “Alzheimer Dementia*” OR “cognitive decline” OR “cognitive impairment” OR “cognitive disorder” OR “cognitive dysfunction” OR “cognition disorder”). The references of the included studies and existing systematic reviews were hand-searched to find additional relevant articles. The full search strategy is presented in Supplementary Table S1 [13].

### 2.2. Study Selection

All selected titles and abstracts were independently reviewed by two authors (Yuanyuan Ye and Yiqing Wang). Studies were excluded if the title and/or abstract were not appropriate for the aim of the review. Full texts were subsequently obtained for eligible studies or when the relevance of an article could not definitively be excluded. Disagreement was resolved by consensus and by opinion of a third reviewer (Shiwei Li), when necessary.

### 2.3. Eligibility Criteria

The included studies were required to meet the following criteria: (1) cohort, case-control, or cross-sectional study; (2) the exposed group consisted of patients with subclinical hypothyroidism or overt hypothyroidism, and the control group consisted of euthyroid participants; (3) participants of 18 years old at least; (4) the risk of dementia or cognitive impairment as the outcome; (5) were published before 4 May 2022; and (6) were published in English.

### 2.4. Exclusion Criteria

Exclusion criteria were as follows: (1) conference abstracts or study protocols; (2) duplicate publications; (3) studies with incomplete data or no relevant outcome; (4) studies that did not include patients with hypothyroidism or subclinical hypothyroidism; (5) studies with no risk of dementia or cognitive impairment as an outcome; and (6) studies involving people with life-threatening cancer, major psychiatric disorders such as major depressive disorder, neurological disorders such as stroke and Parkinson’s disease, or medical disorders that might affect neuropsychological function.

### 2.5. Data Extraction

Two reviewers (Yuanyuan Ye; Yiqing Wang) independently completed the data extraction by using a standardized form. Disagreement was resolved by consensus and the opinion of a third reviewer (Shiwei Li), when required. Detailed information was recorded, including study year, first name of author, country, type of study, sample size, follow-up times, age of participants, diagnosis of hypothyroidism and dementia or cognitive impairment, hypothyroidism type, dementia type, and confounder.

### 2.6. Risk-of-Bias Assessment

The Newcastle–Ottawa Quality Assessment Scale (NOS) [14] was used to assess the quality of the included cohort studies in regard to three aspects, namely selection, comparison, and results. The scores of cohort studies and case-control studies ranged from 0 to 9. Higher scores indicated a higher research quality; specifically, NOS scores of ≥7, 4–6, and 0–3 indicated high, medium, and low quality, respectively. The methodological quality of the included cross-sectional studies was assessed by using the Agency for Healthcare Research and Quality (AHRQ) checklist for observational studies [15]. Scores ranged from 0 to 10, with higher scores indicating higher quality. An overall score ≥ 7 was indicative of good methodological quality across studies, while scores of 1–3 and 4–6 indicated poor and moderate quality, respectively (Supplementary Table S2) [13].

### 2.7. Statistical Analysis

The Stata software (version 14.0) was used to conduct the data analysis. We extracted data on the adjusted OR and 95% CI from each study to assess the risk of dementia or cognitive impairment in patients with hypothyroidism. We assessed heterogeneity by using the chi-square test and I^2^ value, and *p* < 0.1 or I^2^ > 50% was considered to indicate heterogeneity. In such instances, the random-effects model was adopted. Otherwise, the fixed-effects model was employed. Considering the possible heterogeneity caused by differences in hypothyroidism type, cognitive dysfunction type, and research type, we performed subgroup analyses, respectively, according to dementia versus cognitive impairment, overt hypothyroidism versus subclinical hypothyroidism, and prospective studies versus cross-sectional studies. We performed sensitivity analyses to verify the robustness of the overall results. The funnel plots and Egger’s regression test were used to detect the publication bias.

## 3. Results

### 3.1. Literature Search

**Figure 1:**
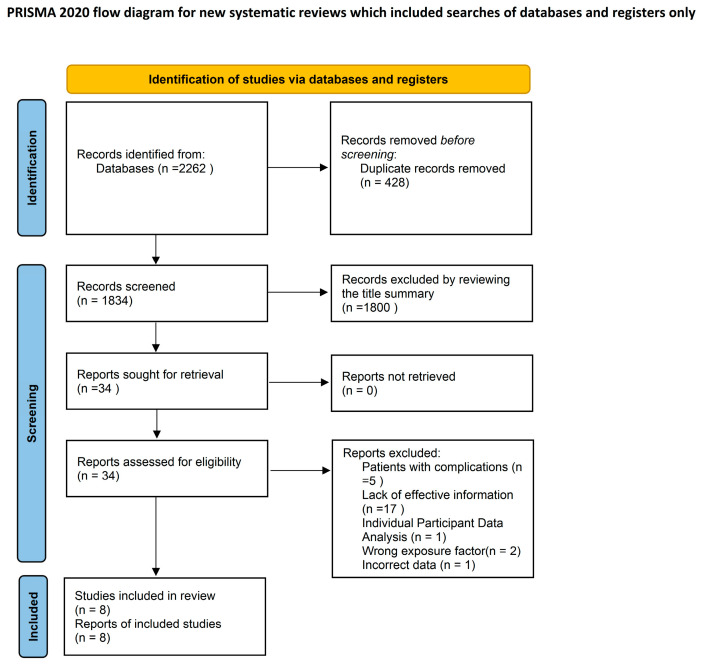
Flow diagram of the literature search and article selection.

The systematic search of relative studies published before 4 May 2022 identified 2262 results. After title and abstract screening, 34 articles were considered potentially relevant. Eight studies [16,17,18,19,20,21,22,23] were included after full-text reviews. The search selection process is shown in Figure 1.

### 3.2. Study Characteristics

This meta-analysis included eight studies [16,17,18,19,20,21,22,23], covering 1,092,025 individuals, and they were published between 2010 and 2021. Twenty-six studies [5,24,25,26,27,28,29,30,31,32,33,34,35,36,37,38,39,40,41,42,43,44,45,46,47,48] were excluded for the following reasons: five studies [25,26,37,43,45] included subjects with complications other than hypothyroidism, seventeen studies [24,27,28,29,30,32,33,34,35,36,38,39,42,44,46,47,48] lacked effective information, one study [5] was an individual-participant data analysis, two studies [31,40] did not meet the inclusion criteria for exposure factors, and one study [41] provided incorrect data. The authors of the last study [41] were consulted to clarify; however, no replies were received. Of the eight studies included in this review, four studies were prospective cohort studies, while the other four were cross-sectional studies. All individuals in these cohorts were at least 18 years old at the beginning of follow-up and had clear diagnostic criteria for dementia or cognitive impairment. The average follow-up time of four prospective cohort studies ranged from 6.2 to 21.9 years. The adjusted estimates were available for almost all studies, even though the adjusted confounders are slightly different. The main characteristics of the included trials are shown in Table 1.

### 3.3. Any Hypothyroidism and Risk of Cognitive Dysfunction

**Figure 2:**
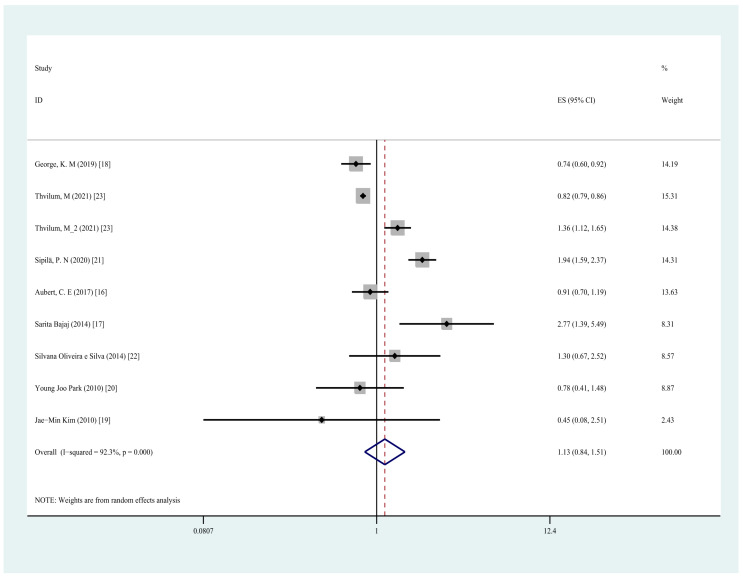
Forest plot of cognitive dysfunction and any type of hypothyroidism.

A total of eight studies [16,17,18,19,20,21,22,23] explored the association between any hypothyroidism and the risk of cognitive dysfunction. The pooling analysis shows that any type of hypothyroidism is not associated with an increased risk of cognitive impairment or dementia (OR = 1.13, 95% CI = 0.84–1.51, I^2^ = 92.3%, *p* = 0.426; Figure 2). A sensitivity analysis showed that none of the individual studies reversed the pooled-effect size, meaning that the results are robust. The sensitivity analysis is included in Supplementary Figure S1 [13].

### 3.4. Any Hypothyroidism and Risk of Dementia

**Figure 3:**
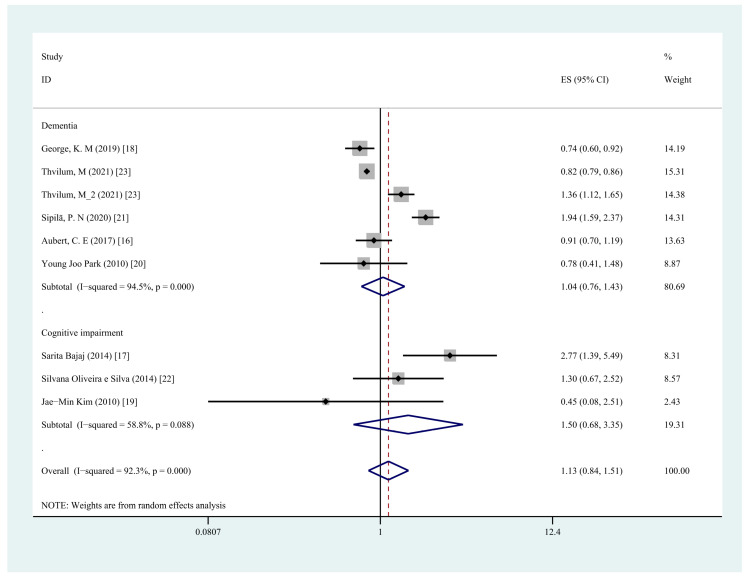
Forest plot of cognitive impairment vs. dementia and any type of hypothyroidism.

Five studies [16,18,20,21,23] assessed the association between any type of hypothyroidism and the risk of dementia. The pooled results showed that any type of hypothyroidism is not associated with an increased risk of dementia (OR = 1.04, 95% CI = 0.76–1.43, I^2^ = 94.5%, *p* = 0.80; Figure 3).

### 3.5. Any Hypothyroidism and Risk of Cognitive Impairment

Three studies [17,19,22] assessed the association between any type of hypothyroidism and the risk of cognitive impairment. The pooled results showed that any type of hypothyroidism is not associated with an increased risk of cognitive impairment (OR = 1.50, 95% CI = 0.68–3.35, I^2^ =58.8%, *p* = 0.318; Figure 3).

### 3.6. Subgroup Analysis

**Figure 4:**
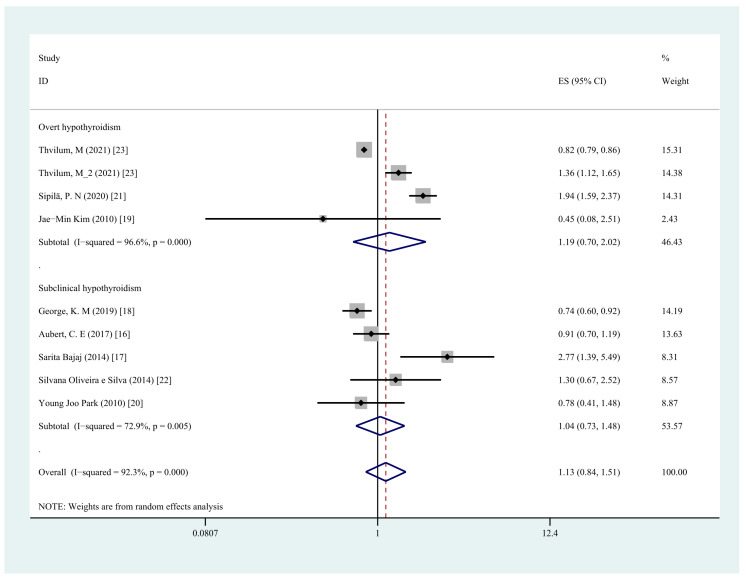
Forest plot of overt hypothyroidism vs. subclinical hypothyroidism and cognitive dysfunction.

**Figure 5:**
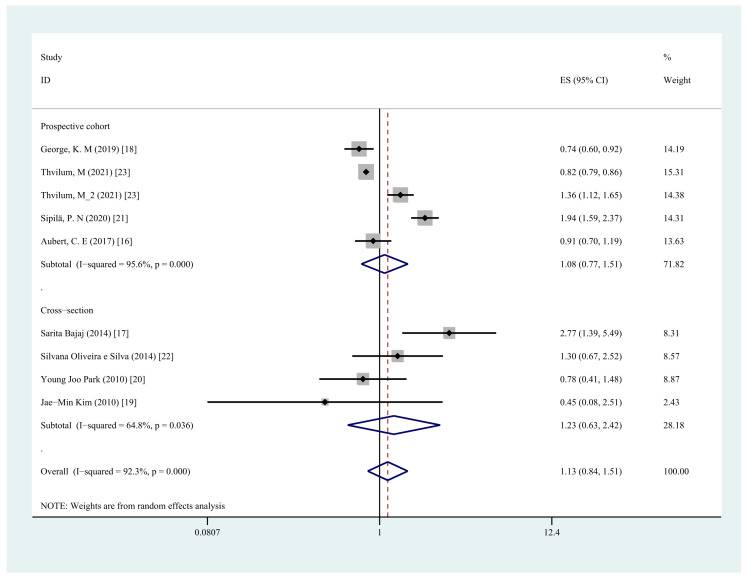
Forest plots depicting the associations observed between any type of hypothyroidism and cognitive dysfunction in prospective cohort vs. cross-section studies.

**Figure 6:**
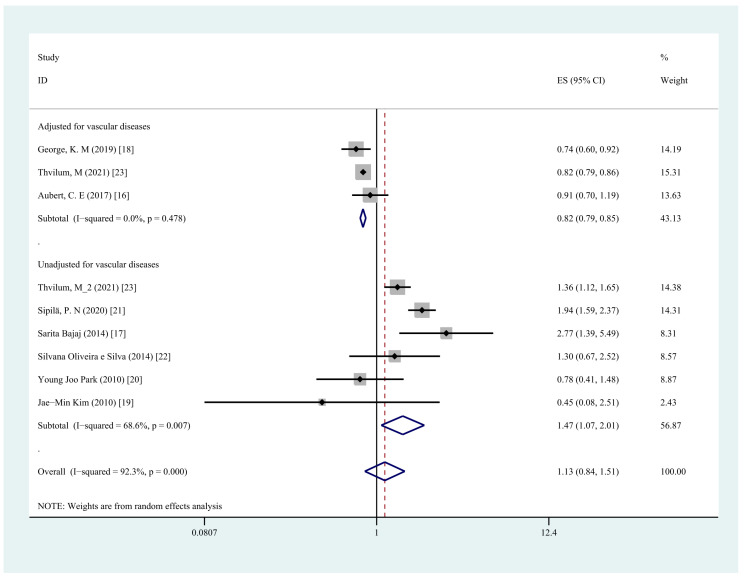
Forest plots depicting the associations observed between any type of hypothyroidism and cognitive dysfunction in studies adjusted for vascular diseases vs. studies unadjusted for vascular diseases.

We conducted a subgroup analysis of hypothyroidism type, research type, and adjustment of comorbidities, and the first two analyses did not find the origin of the high heterogeneity, while significant results were obtained by the analysis of comorbidity adjustment. In the subgroup analysis, neither overt hypothyroidism (OR = 1.19, 95% CI = 0.70–2.02, I2 = 96.6%, *p* = 0.525) nor subclinical hypothyroidism (OR = 1.04, 95% CI = 0.73–1.48, I^2^ = 72.9%, *p* = 0.833) was associated with an increased risk of cognitive dysfunction (Figure 4). The subgroup analyses by type of study design showed a similar trend in the prospective cohort studies group (OR = 1.08, 95% CI = 0.77–1.51, I^2^ = 95.6%, *p* = 0.673) compared with the cross-sectional studies (OR = 1.23, 95% CI = 0.63–2.42, I^2^ = 64.8%, *p* = 0.545) (Figure 5). Subgroup analyses by comorbidity adjustment showed that the risk of cognitive dysfunction was significantly increased in the group not adjusted for vascular comorbidity (OR = 1.47, 95% CI = 1.07–2.01, I^2^ = 68.6%, *p* = 0.017), whereas the risk was decreased in the group adjusted for comorbidities, including vascular diseases (OR = 0.82, 95% CI = 0.79–0.85, I^2^ = 0.0%, *p* < 0.001) (Figure 6 and Table 2).

### 3.7. Publication Bias

**Figure 7:**
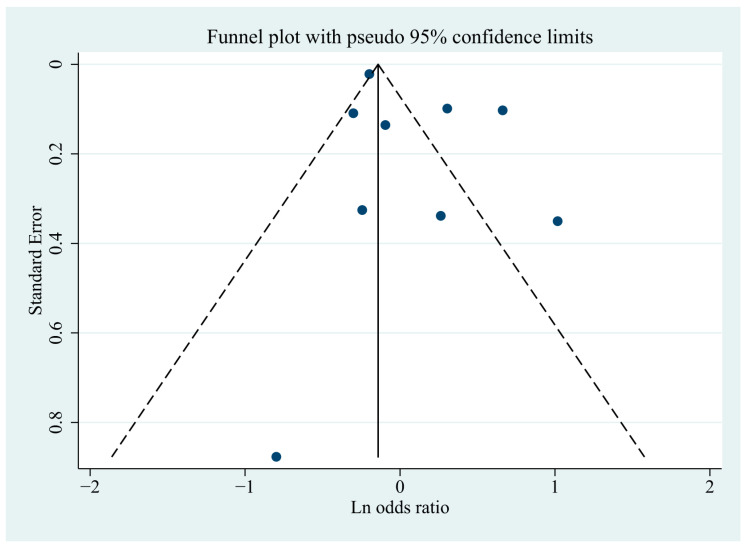
Funnel plot of standard errors by lnOR.

A visual inspection of the funnel plot showed no evidence of a significant publication bias in the outcome of any hypothyroidism and risk of cognitive dysfunction (Figure 7). Egger’s regression test (*p* = 0.171), likewise, indicated no publication bias in our meta-analysis.

## 4. Discussion

In this meta-analysis of eight studies [16,17,18,19,20,21,22,23], including two different thyroid dysfunctions (subclinical hypothyroidism and overt hypothyroidism), without considering comorbidities, we did not find evidence supporting an association between any hypothyroidism with cognitive dysfunction. When we performed a subgroup analysis by hypothyroidism type and study type, we also did not obtain meaningful results. However, in the subgroup analysis of comorbidities, including vascular diseases, we found a contrary effect of hypothyroidism on cognitive dysfunction. The eight studies [16,17,18,19,20,21,22,23] included four prospective studies and four cross-sectional studies. Of the four cross-sectional studies [17,19,20,22], only one study [17] that included 103 subclinical hypothyroidism cases and 103 controls concluded that the prevalence of cognitive impairment was significantly higher in subclinical hypothyroidism. The four prospective studies [16,18,21,23] included in this analysis report five prospective cohorts and draw conflicting conclusions, with sample sizes ranging from 2558 to 557,825. Two cohorts [18,23] concluded that patients with overt hypothyroidism or subclinical hypothyroidism were at a reduced risk of dementia after adjustment for comorbidities, including cardiovascular disease. One cohort [23] focused on overt hypothyroidism, and after adjustment for the Charlson Comorbidity Index, the association between hypothyroidism and dementia was significantly reduced (HR = 0.82; 95% CI: 0.79–0.86). The other cohort [18] focused on subclinical hypothyroidism, and after adjusting for comorbidities, including cardiovascular disease, subclinical hypothyroidism was associated with a lower risk of dementia (HR = 0.74; 95% CI: 0.60–0.92). Two cohorts [21,23] concluded that hypothyroid patients had an increased risk of dementia. The OPENTHYRO cohort [23] concluded that every 6-month increase in TSH was associated with a significantly increased risk of dementia (HR = 1.12; 95% CI: 1.07–1.16). Another exploratory multi-cohort study [21] of 283,414 community-dwelling participants concluded that the risk of dementia was significantly increased with hypothyroidism (HR = 1.94; 95% CI: 1.59–2.38). There was also a prospective population-based cohort study [16] that included 2558 participants aged 70–79 years that concluded that subclinical hypothyroidism was not significantly associated with dementia (HR = 0.91, 95% CI: 0.70–1.19).

Two recent meta-analyses [6,11] are consistent with our findings; one [11] focused on the association between serum thyroxine, thyrotropin, and dementia, showing no significant association between dementia and high TSH levels. A stratified analysis by case-control studies showed a protective tendency of high TSH levels against dementia, consistent with the result of the DNPR cohort included in this meta-analysis, showing an attenuated risk of dementia with hypothyroidism after adjustment for preexisting comorbidity (HR 0.82; 95% CI: 0.79–0.86). Another study [6] of people over 60 years old provides no evidence to support an association between subclinical hypothyroidism and cognitive impairment in relatively healthy older adults (pooled estimate (ES) 0.03; 95% CI: 0.001–0.067). A large cross-sectional community study [30] of subjects over 65 years of age showed that subclinical hypothyroidism was not associated with cognitive function. The clock mapping test was used in this study to measure cognitive status, and no significant differences were found in cognitive status between the case and control groups. In addition, a recently published review [49] showed no improvement in hypothyroid symptoms, cardiac, and skeletal parameters after levothyroxine treatment in elderly patients with subclinical hypothyroidism. These data suggest that treatment with levothyroxine should be considered in patients 65 years of age or older with subclinical hypothyroidism when thyroid stimulating hormone concentrations are consistently 7 mIU/L or higher, rather than starting treatment when thyroid stimulating hormone concentrations are below 7 mIU/L. The dose of levothyroxine should be individualized based on age, comorbidities, and life expectancy.

However, there are some studies [50,51,52] that have reached different conclusions from us. A study [50] of 1047 British subjects aged ≥64 years showed a correlation between TSH values and cognitive performance. However, this conclusion was drawn from the study’s analysis of the baseline data. This study also came to the interesting conclusion that high normal FT4 levels were independently associated with accelerated cognitive decline in people without significant thyroid disease. Other studies [51] have found that thyroxine produces oxidative stress and damages neurons, suggesting that, perhaps, in the future, we can focus our attention on FT4 rather than TSH. In a recently published REVIEW study [53], the authors suggested that chronic exposure to thyroid hormones can lead to cardiovascular diseases, such as systolic hypertension and atrial fibrillation, associated with an increased risk of dementia. However, this study also mentions that compensatory normal thyroid function status does not seem to restore all aspects of cognitive impairment caused by thyroid dysfunction. Parle et al. [52] found no difference between the L-T4 and placebo groups on any cognitive measure. It has also been suggested [54] that there is a physiological increase in TSH toward the upper limit during normal aging. Therefore, based on these data, it is again proven that thyroxine treatment is not recommended for people without thyroid disease. However, this does not mean that patients with subclinical thyroid dysfunction or normal thyroid function should be ignored. Studies [55,56] on patients with hormonally compensated autoimmune thyroiditis with normal thyroid function suggested the presence of central nervous system impairment even during the compensated thyroid function phase. Vascular inflammatory changes and cytotoxic brainstem edema during the disease process are non-negligible causes of the progressive development of cognitive impairment.

Thyroid hormones have many physiological effects and have important implications for cerebral blood flow. One study [57] found reduced temporal lobe and thalamic blood flow in patients with subclinical hypothyroidism, and this reduction in cerebral perfusion appears to be specific to brain regions associated with memory [58]. This suggests to us the importance of cardiovascular disease in the association between thyroid dysfunction and cognitive impairment. Both hypothyroidism [59] and cognitive dysfunction [60] have multifactorial etiologies; when the effect of hypothyroidism on cognitive dysfunction is diluted by a higher burden of comorbidity, it is important to consider whether the association is indirect and to look harder for common exposures. A Brazilian study [61] on subclinical hyperthyroidism and dementia suggested that the association between cardiovascular disease and cognitive impairment needs to be taken seriously. The cardiovascular burden was at the top of the list for the people included in the study. Moreover, the study did not find positive results in the analysis of the association between subclinical hypothyroidism and dementia, as is consistent with our study. When cardiovascular comorbidities were taken into account, after excluding subjects with heart failure and cerebrovascular accidents, the study by Agarwal Rachna et al. [62] suggested that an increase in TSH reduced the odds of dementia.

It is reported [63] that elevated exosomal ApoE4 demonstrated a significant inverse correlation with the serum level of thyroid hormones and cognitive function. It is thyroid hormone levels, not thyroid-stimulating hormone levels, that are associated with cognition dysfunction, so is it possible to speculate that age-related TSH elevations are a form of compensation to maintain normal thyroid hormone levels to ensure normal cognitive function requirements? Perhaps this could explain the conclusion reached in some studies that aging-related subclinical hypothyroidism is a protective factor for cognitive impairment in the elderly.

Reference [63] suggested a possible mechanism for the relationship between hypothyroidism and cognitive impairment. Ageing-associated hypothyroidism and acute thyroidectomy increased the transport of liver-derived ApoE4 to exosomes and into the brain, where ApoE4 activated the nucleotide-binding oligomerization domain-like receptor family pyrin domain-containing 3 (NLRP3) inflammasome by increasing the cholesterol level in neural cells. This, in turn, affected cognition, locomotion, and mood.

The fact that the eight studies we included used different diagnostic criteria to diagnose hypothyroidism and cognitive impairment may be one of the reasons why no significant results were obtained in this study. What is more, since seven of our included studies relied on thyroid hormone and thyroid-stimulating hormone concentrations to arrive at the diagnosis, the consistency of the timing and method of hormone testing can also have an impact on results. It has been noted [58] that patients with Alzheimer’s disease have dysregulated thyroid hormone levels and circadian rhythms. Therefore, the timing of sample collection and fasting status are important, especially considering that small changes in thyroid hormones can lead to the misclassification of the patient’s thyroid status. The fact that the included studies used different assays and that only two studies stated the timing of the assay may also contribute to inconsistency in hormone testing (Table 1).

However, we found a decreased risk of cognitive dysfunction in hypothyroidism participants in studies adjusting for pre-existing morbidity, including vascular diseases. This result suggested that comorbidities, including cardiovascular diseases, may mediate the risk of hypothyroidism and cognitive dysfunction. Moreover, overtreatment is associated with an increased risk of atrial fibrillation and atherosclerosis and, through cerebrovascular injury, may be associated with an increased risk of cognitive decline. Additional large prospective cohort studies and other observational studies are still needed to determine the cognitive outcomes in hypothyroidism patients with different complications.

Dementia is a growing clinical and socioeconomic problem in an aging society. Furthermore, subclinical hypothyroidism or overt hypothyroidism can harm health and quality of life. Although the mechanism of the association between hypothyroidism and cognitive dysfunction is not precise, it is still essential to focus on the prevention of hypothyroidism. Modifying iodine deficiency [64] or iodine excess [65], nutraceuticals such as omega-3 PUFA supplementation [66], and improving sleep quality [67] all have positive implications. Accurate assessment of whether hypothyroidism is associated with an increased risk of dementia or cognitive impairment is important for the accurate prevention and treatment of dementia. Thus, for patients with different diseases, the association between overt or subclinical hypothyroidism and cognitive impairment remains to be explored.

Although bias may exist due to the observational nature of the included studies and the different data collection methods (prospective or retrospective), the relatively large combined sample size and the good quality of the included studies may enhance the power of the analysis.

## 5. Conclusions

Our meta-analysis shows that hypothyroidism was not associated with risk of cognitive dysfunction without distinguishing comorbidities. Hypothyroidism was associated with a reduced risk of cognitive dysfunction after adjustment for vascular disease comorbidities. More prospective observational studies are needed in the future.

## Figures and Tables

**Table 1:** The main characteristics of the included studies.

Authors	Country	Study Type	Sample Size	Follow-Up Years	Age (Years)	Diagnosis of Hypothyroidism	Hormone Test Methods and Sample Collection Time	Diagnosis of Dementia/Cognitive Impairment	Hypothyroidism Type	Dementia Type	Confounders Adjusted
George et al., 2019 [18]	USA	Prospective cohort	12,481	21.9	57 ± 5.7	TSH and FT4 concentration	Elecsys 2010 analyzer, a sandwich immunoassay method for TSH and competition immunoassay method for FT4; Time: NA	Cognitive test performance, neuropsychological examinations, clinician review of suspected cases, telephone interviews, relevant hospital, and death-certificate codes	Overt hypothyroidism, subclinical hypothyroidism	MCI, dementia	Age, sex, race-center, APOE ε4, income and education, BMI, smoking status, hypertension, diabetes, drinking status, HDL-C and total cholesterol, prevalent CVD, and baseline thyroid medication use
Thvilum et al., 2021 [23]	Denmark	Prospective cohort	557,825	6.2	55.8 (43.3–68.5)	ICD-10 codes: E03.2-E03.9; ATC code: H03A	NA	ICD-10 codes; ATC codes	Hypothyroidism	Dementia, Alzheimer’s disease, vascular dementia, and other forms of dementia	Charlson Comorbidity Index
Thvilum et al., 2021 [23]	Denmark	Prospective cohort	233,844	7.2	Hypothyroid Individuals: 56.4 (42.6–69.2); reference Individuals: 50.3 (36.4–64.2)	TSH concentration	NA	ICD-10 codes	Hypothyroidism	Dementia	NA
Sipilä et al., 2020 [21]	Finland	Prospective cohort	283,414	19	33.5 (18.0–87.9)	ICD-10 codes: E03	NA	ICD-10 codes	Hypothyroidism	Dementia	Age, sex, low education/socioeconomic status, hypertension, smoking, depression, physical inactivity, diabetes, marital status (a proxy for social isolation), obesity (available in all cohorts except STW), and APOE genotype (available in WHII)
Aubert et al., 2017 [16]	Switzerland	Prospective cohort	2558	over 10 years	75.1 (2.8)	TSH and FT4 concentration	ACS; Chiron Diagnostics Corp, Emeryville, Calif, immunoassay for TSH and competitive immunoassay for FT4; Time: NA	Application of 3 MS score, diagnosis of hospitalization or prescription of dementia drug	Subclinical hypothyroidism	Dementia	Age, sex, race, education, and baseline 3 MS, and then further for cardiovascular risk factors
Bajaj et al., 2014 [17]	India	Cross-section	206	-	Cases:75.74 ± 9.37; Controls:75.72 ± 9.40	TSH, FT3 and FT4 concentration	Immulite-1000 TSH, chemiluminescent detection for TSH; Time: NA	MMSE test and The Clock Drawing Test	Subclinical hypothyroidism	Cognitive function	NA
SO, E.S., et al., 2014 [22]	Brazil	Cross-section	284	-	80.7 ± 6.7	TSH and FT4 concentration	Immulite 2000^®^, chemiluminescence for TSH and FT4; Time: at morning	MMSE test	Subclinical hypothyroidism	Cognitive deficit	NA
Park et al., 2010 [20]	Korea	Cross-section	918	-	Subclinical hypothyroidism: 76.5 ± 9.0; euthyroidism: 76.8 ± 9.0	TSH and FT4 concentration	Immunoradiometric assays (TSH: CIS bio international, Gif-sur-Yvette, France; FT4: DiaSorin S.p.A, Saluggia, Italy); Time: NA	DSM-IV diagnostic criteria	Subclinical hypothyroidism	Dementia	Age, gender, and duration of education
Kim et al., 2010 [19]	Korea	Cross-section	495	-	72.4 (5.6)	TSH concentration	Chemiluminescent immunoassay (Cobas: Roche Diagnostics, West Sussex, UK) for TSH; Time: at morning	CSID test	Hypothyroidism	Cognitive impairment	Age, gender, education, smoking, physical activity, systolic BP, diabetes mellitus, total cholesterol, albumin, levothyroxine treatment, and depression

Abbreviations: TSH, thyroid-stimulating hormone; FT4, free thyroxine; MCI, Mild cognitive impairment; APOE ε4, apolipoprotein E ε4; BMI, body mass index; HDL-C, high density lipoprotein cholesterol; CVD, cardiovascular disease; ICD-10 codes, International Classification of Diseases 10th revision codes; ATC, Anatomical Therapeutic Chemical Classification System; NA, not available; STW, Still Working study; WHII, Whitehall II study; 3 MS, Modified Mini-Mental State Examination;FT3, free triiodothyronine; MMSE, Mini-Mental State Examination; DSM-IV diagnostic criteria, Diagnostic and Statistical Manual of Mental Disorders; CSID, Community Screening Interview for Dementia.

**Table 2:** Subgroup analysis for the risk of cognitive dysfunction in patients with hypothyroidism.

Subgroups	Included Studies	OR (95% CI)	Heterogeneity	
			I^2^ (%)	*p*-Values
Hypothyroidism Type				
Overt hypothyroidism	4	1.19 (0.70,2.02)	96.6%	0.53
Subclinical hypothyroidism	5	1.04 (0.73,1.48)	72.9%	0.83
Research type				
Prospective cohort	5	1.08 (0.77,1.51)	95.6%	0.67
Cross-section	4	1.23 (0.63,2.42)	64.8%	0.55
Adjustment of comorbidities				
Adjusted for vascular diseases	3	0.82 (0.79,0.85)	0.0%	<0.001
Unadjusted for vascular diseases	6	1.47 (1.07,2.01)	68.6%	0.02

## Data Availability

The original contributions presented in the study are included in the article/Supplementary Materials [13]; further inquiries can be directed to the corresponding authors.

## Supplementary Materials

The following supporting information can be downloaded at https://www.mdpi.com/article/10.3390/jcm11226726/s1. Table S1: Details of the literature search strategy. Table S2: Newcastle–Ottawa Quality Assessment Scale and Agency for Healthcare Research and Quality scale for cohort studies and cross-sectional studies included in this review. Table S3: Preferred Reporting Items for Systematic Reviews and Meta-Analyses (PRISMA) checklist. Figure S1: Sensitivity analysis of the risk of cognitive dysfunction caused by any type of hypothyroidism.

## References

[1] Burns A., Iliffe S. (2009). Dementia. BMJ.

[2] Prince M., Wimo A., Guerchet M., Ali G.C., Prina M. (2015). World Alzheimer Report 2015. The Global Impact of Dementia. An Analysis of Prevalence, Incidence, Cost and Trends.

[3] Petersen R.C., Roberts R.O., Knopman D.S., Boeve B.F., Geda Y.E., Ivnik R.J., Smith G.E., Jack C.R. (2009). Mild Cognitive Impairment. Arch. Neurol..

[4] Waldemar G., Dubois B., Emre M., Georges J., McKeith I., Rossor M., Scheltens P., Tariska P., Winblad B. (2007). Recommendations for the diagnosis and management of Alzheimer’s disease and other disorders associated with dementia: EFNS guideline. Eur. J. Neurol..

[5] Van Vliet N.A., van Heemst D., Almeida O.P., Åsvold B.O., Aubert C.E., Bin Bae J., Barnes L.E., Bauer D.C., Blauw G.J., Brayne C. (2021). Association of Thyroid Dysfunction with Cognitive Function. JAMA Intern. Med..

[6] Akintola A.A., Jansen S.W., Bodegom D.E., Der Grond J.E., Westendorp R.G., De Craen A.J.M., Heemst D.E. (2015). Subclinical hypothyroidism and cognitive function in people over 60 years: A systematic review and meta-analysis. Front. Aging Neurosci..

[7] Joffe R.T., Pearce E.N., Hennessey J.V., Ryan J.J., Stern R. (2012). Subclinical hypothyroidism, mood, and cognition in older adults: A review. Int. J. Geriatr. Psychiatry.

[8] Parsaik A.K., Singh B., Roberts R.O., Pankratz S., Edwards K.K., Geda Y.E., Gharib H., Boeve B.F., Knopman D.S., Petersen R.C. (2014). Hypothyroidism and Risk of Mild Cognitive Impairment in Elderly Persons. JAMA Neurol..

[9] Pasqualetti G., Pagano G., Rengo G., Ferrara N., Monzani F. (2015). Subclinical Hypothyroidism and Cognitive Impairment: Systematic Review and Meta-Analysis. J. Clin. Endocrinol. Metab..

[10] Rieben C., Segna D., da Costa B.R., Collet T.-H., Chaker L., Aubert C.E., Baumgartner C., Almeida O., Hogervorst E., Trompet S. (2016). Subclinical Thyroid Dysfunction and the Risk of Cognitive Decline: A Meta-Analysis of Prospective Cohort Studies. J. Clin. Endocrinol. Metab..

[11] Wu Y., Pei Y., Wang F., Xu D., Cui W. (2016). Higher FT4 or TSH below the normal range are associated with increased risk of dementia: A meta-analysis of 11 studies. Sci. Rep..

[12] Page M.J., McKenzie J.E., Bossuyt P.M., Boutron I., Hoffmann T.C., Mulrow C.D., Shamseer L., Tetzlaff J.M., Akl E.A., Brennan S.E. (2021). The PRISMA 2020 Statement: An Updated Guideline for Reporting Systematic Reviews. BMJ.

[13] Liu M., Ding L. (2022). Association of Hypothyroidism and the Risk of Cognitive Dysfunction: A Meta-Analysis [Internet]. Figshare.

[14] Stang A. (2010). Critical evaluation of the Newcastle-Ottawa scale for the assessment of the quality of nonrandomized studies in meta-analyses. Eur. J. Epidemiol..

[15] Kaptein S., Geertzen J.H., Dijkstra P.U. (2017). Association between cardiovascular diseases and mobility in persons with lower limb amputation: A systematic review. Disabil. Rehabil..

[16] Aubert C.E., Bauer D.C., da Costa B.R., Feller M., Rieben C., Simonsick E.M., Yaffe K., Rodondi N. (2017). The Health ABC Study The association between subclinical thyroid dysfunction and dementia: The Health, Aging and Body Composition (Health ABC) Study. Clin. Endocrinol..

[17] Bajaj S., Sachan S., Misra V., Varma A., Saxena P. (2014). Cognitive function in subclinical hypothyroidism in elderly. Indian J. Endocrinol. Metab..

[18] George K., Lutsey P.L., Selvin E., Palta P., Windham B.G., Folsom A.R. (2019). Association Between Thyroid Dysfunction and Incident Dementia in the Atherosclerosis Risk in Communities Neurocognitive Study. J. Endocrinol. Metab..

[19] Kim J.-M., Stewart R., Kim S.-Y., Bae K.-Y., Yang S.-J., Kim S.-W., Shin I.-S., Yoon J.-S. (2010). Thyroid Stimulating Hormone, Cognitive Impairment and Depression in an Older Korean Population. Psychiatry Investig..

[20] Park Y.J., Lee E.J., Lee Y.J., Choi S.H., Park J.H., Lee S.B., Lim S., Lee W.W., Jang H.C., Cho B.Y. (2010). Subclinical hypothyroidism (SCH) is not associated with metabolic derangement, cognitive impairment, depression or poor quality of life (QoL) in elderly subjects. Arch. Gerontol. Geriatr..

[21] Sipilä P.N., Lindbohm J.V., Singh-Manoux A., Shipley M.J., Kiiskinen T., Havulinna A.S., Vahtera J., Nyberg S.T., Pentti J., Kivimäki M. (2020). Long-term risk of dementia following hospitalization due to physical diseases: A multicohort study. Alzheimer’s Dement..

[22] E Silva S.O., Chan I.T., Lobo Santos M.A., Cohen M., de La Roque P Araujo M., da Silva Almeida J., Simões A., Givigi H.R.B., Vaisman M., Paixão C.M. (2014). Impact of thyroid status and age on comprehensive geriatric assessment. Endocrine.

[23] Thvilum M., Brandt F., Lillevang-Johansen M., Folkestad L., Brix T.H., Hegedüs L. (2021). Increased risk of dementia in hypothyroidism: A Danish nationwide register-based study. Clin. Endocrinol..

[24] Tappy L., Randin J.P., Schwed P., Wertheimer J., Lemarchand-Béraud T. (1987). Prevalence of thyroid disorders in psychogeriatric inpatients. A possible relationship of hypothyroidism with neurotic depression but not with dementia. J. Am. Geriatr. Soc..

[25] Percy M.E., Dalton A.J., Markovic V.D., McLachlan D.R.C., Gera E., Hummel J.T., Rusk A.C.M., Somerville M.J., Andrews D.F., Walfish P.G. (1990). Autoimmune thyroiditis associated with mild “subclinical” hypothyroidism in adults with down syndrome: A comparison of patients with and without manifestations of Alzheimer disease. Am. J. Med Genet..

[26] Bhaumik S., Collacott R.A., Garrick P., Mitchell C. (1991). Effect of thyroid stimulating hormone on adaptive behaviour in Down’s syndrome. J. Intellect. Disabil. Res..

[27] Osterweil D., Syndulko K., Cohen S.N., Hershman J.M., Cummings J.L., Tourtellotte W.W., Solomon D.H., Pettier-Jennings P.D. (1992). Cognitive Function in Non-Demented Older Adults with Hypothyroidism. J. Am. Geriatr. Soc..

[28] Volpato S., Guralnik J.M., Fried L.P., Remaley A.T., Cappola A.R., Launer L.J. (2002). Serum thyroxine level and cognitive decline in euthyroid older women. Neurology.

[29] Münte T.F., Lill C., Ötting G., Brabant G. (2004). Cognitive changes in short-term hypothyroidism assessed with event-related brain potentials. Psychoneuroendocrinology.

[30] Roberts L.M., Pattison H., Roalfe A., Franklyn J., Wilson S., Hobbs F.R., Parle J.V. (2006). Is Subclinical Thyroid Dysfunction in the Elderly Associated with Depression or Cognitive Dysfunction?. Ann. Intern. Med..

[31] Suhanov A.V., Pilipenko P.I., Korczyn A.D., Hofman A., Voevoda M.I., Shishkin S.V., Simonova G.I., Nikitin Y.P., Feigin V.L. (2006). Risk factors for Alzheimer’s disease in Russia: A case–control study. Eur. J. Neurol..

[32] Tan Z.S. (2008). Thyroid Function and the Risk of Alzheimer DiseaseThe Framingham Study. Arch. Intern. Med..

[33] Ceresini G., Lauretani F., Maggio M., Ceda G.P., Morganti S., Usberti E., Chezzi C., Valcavi R., Bandinelli S., Guralnik J.M. (2009). Thyroid Function Abnormalities and Cognitive Impairment in Elderly People: Results of the Invecchiare in Chianti Study. J. Am. Geriatr. Soc..

[34] Correia N., Mullally S., Cooke G., Tun T.K., Phelan N., Feeney J., FitzGibbon M., Boran G., O’Mara S., Gibney J. (2009). Evidence for a Specific Defect in Hippocampal Memory in Overt and Subclinical Hypothyroidism. J. Clin. Endocrinol. Metab..

[35] Resta F., Triggiani V., Barile G., Benigno M., Suppressa P., Giagulli V.A., Guastamacchia E., Sabba C. (2012). Subclinical hypothyroidism and cognitive dysfunction in the elderly. Endocrine, Metab. Immune Disord. Drug Targets.

[36] Yamamoto N., Ishizawa K., Ishikawa M., Yamanaka G., Yamanaka T., Murakami S., Hiraiwa T., Okumiya K., Ishine M., Matsubayashi K. (2012). Cognitive function with subclinical hypothyroidism in elderly people without dementia: One year follow up. Geriatr. Gerontol. Int..

[37] Wijsman L.W., De Craen A.J.M., Trompet S., Gussekloo J., Stott D.J., Rodondi N., Welsh P., Jukema J.W., Westendorp R.G.J., Mooijaart S.P. (2013). Subclinical Thyroid Dysfunction and Cognitive Decline in Old Age. PLoS ONE.

[38] Formiga F., Ferrer A., Padros G., Contra A., Corbella X., Pujol R. (2014). Thyroid status and functional and cognitive status at baseline and survival after 3 years of follow-up: The OCTABAIX study. Eur. J. Endocrinol..

[39] Paladugu S., Hanmayyagari B.R., Kudugunti N., Reddy R., Sahay R., Ramesh J. (2015). Improvement in subclinical cognitive dysfunction with thyroxine therapy in hypothyroidism: A study from tertiary care center. Indian J. Endocrinol. Metab..

[40] Hu Y., Wang Z.-C., Guo Q.-H., Cheng W., Chen Y.-W. (2016). Is thyroid status associated with cognitive impairment in elderly patients in China?. BMC Endocr. Disord..

[41] Juárez-Cedillo T., Basurto-Acevedo L., Vega-García S., Martha A.S.-R., Retana-Ugalde R., Roberto C.G.-M., Escobedo-De-La-Peña J. (2017). Prevalence of thyroid dysfunction and its impact on cognition in older mexican adults: (SADEM study). J. Endocrinol. Investig..

[42] Szlejf C., Suemoto C.K., Santos I.S., Lotufo P.A., Diniz M.D.F.H.S., Barreto S.M., Benseñor I.M. (2018). Thyrotropin level and cognitive performance: Baseline results from the ELSA-Brasil Study. Psychoneuroendocrinology.

[43] Wändell P., Carlsson A.C., Sundquist J., Sundquist K. (2019). Effect of Levothyroxine Treatment on Incident Dementia in Adults with Atrial Fibrillation and Hypothyroidism. Clin. Drug Investig..

[44] Kalra P., Kumaraswamy D.R., Dharmalingam M., Saini J., Yadav R. (2020). Neuropsychological Impairments in Young Patients with Subclinical Hypothyroidism: A Case Control Study. Ann. Neurosci..

[45] Lai F., Mercaldo N.D., Wang C.M., Hersch M.S., Hersch G.G., Rosas H.D. (2021). Association between Hypothyroidism Onset and Alzheimer Disease Onset in Adults with Down Syndrome. Brain Sci..

[46] Mulat B., Ambelu A., Yitayih S., Gela Y.Y., Adera A., Yeshaw Y., Akalu Y. (2021). Cognitive Impairment and Associated Factors Among Adult Hypothyroid Patients in Referral Hospitals, Amhara Region, Ethiopia: Multicenter Cross-Sectional Study. Neuropsychiatr. Dis. Treat..

[47] Szlejf C., Suemoto C.K., Janovsky C.C.P.S., Bertola L., Barreto S.M., Lotufo P.A., Benseñor I.M. (2021). Subtle Thyroid Dysfunction Is Not Associated with Cognitive Decline: Results from the ELSA-Brasil. J. Alzheimers Dis..

[48] Kim J.H., Lee H.S., Kim Y.H., Kwon M.J., Kim J.-H., Min C.Y., Yoo D.M., Choi H.G. (2022). The Association Between Thyroid Diseases and Alzheimer’s Disease in a National Health Screening Cohort in Korea. Front. Endocrinol..

[49] Biondi B., Cappola A.R. (2022). Subclinical hypothyroidism in older individuals. Lancet Diabetes Endocrinol..

[50] Hogervorst E., Huppert F., Matthews F., Brayne C. (2008). Thyroid function and cognitive decline in the MRC Cognitive Function and Ageing Study. Psychoneuroendocrinology.

[51] Cheng P.-K., Chen H.-C., Kuo P.-L., Chang J.-W., Chang W.-T., Huang P.-C. (2022). Associations between Oxidative/Nitrosative Stress and Thyroid Hormones in Pregnant Women–Tainan Birth Cohort Study (TBCS). Antioxidants.

[52] Parle J., Roberts L., Wilson S., Pattison H., Roalfe A., Haque M.S., Heath C., Sheppard M., Franklyn J., Hobbs F.D.R. (2010). A Randomized Controlled Trial of the Effect of Thyroxine Replacement on Cognitive Function in Community-Living Elderly Subjects with Subclinical Hypothyroidism: The Birmingham Elderly Thyroid Study. J. Clin. Endocrinol. Metab..

[53] Khaleghzadeh-Ahangar H., Talebi A., Mohseni-Moghaddam P. (2022). Thyroid Disorders and Development of Cognitive Impairment: A Review Study. Neuroendocrinology.

[54] Boucai L., Hollowell J.G., Surks M.I. (2011). An Approach for Development of Age-, Gender-, and Ethnicity-Specific Thyrotropin Reference Limits. Thyroid.

[55] Waliszewska-Prosół M., Bladowska J., Budrewicz S., Sąsiadek M., Dziadkowiak E., Ejma M. (2021). The evaluation of Hashimoto’s thyroiditis with event-related potentials and magnetic resonance spectroscopy and its relation to cognitive function. Sci. Rep..

[56] Waliszewska-Prosół M., Ejma M. (2021). Assessment of Visual and Brainstem Auditory Evoked Potentials in Patients with Hashimoto’s Thyroiditis. J. Immunol. Res..

[57] Haji M., Kimura N., Hanaoka T., Aso Y., Takemaru M., Hirano T., Matsubara E. (2015). Evaluation of Regional Cerebral Blood Flow in Alzheimer’s Disease Patients with Subclinical Hypothyroidism. Dement. Geriatr. Cogn. Disord..

[58] Figueroa P.B.S., Ferreira A.F.F., Britto L.R., Doussoulin A.P., Torrão A.D.S. (2021). Association between thyroid function and Alzheimer’s disease: A systematic review. Metab. Brain Dis..

[59] Brix T.H., Kyvik K.O., Hegedüs L. (2000). A Population-Based Study of Chronic Autoimmune Hypothyroidism in Danish Twins 1. J. Clin. Endocrinol. Metab..

[60] Beydoun M.A., Beydoun H.A., Gamaldo A.A., Teel A., Zonderman A.B., Wang Y. (2014). Epidemiologic studies of modifiable factors associated with cognition and dementia: Systematic review and meta-analysis. BMC Public Health.

[61] Benseñor I.M., A Lotufo P., Menezes P.R., Scazufca M. (2010). Subclinical hyperthyroidism and dementia: The Sao Paulo Ageing & Health Study (SPAH). BMC Public Heal..

[62] Agarwal R., Kushwaha S., Chhillar N., Kumar A., Dubey D., Tripathi C.B. (2013). A cross-sectional study on thyroid status in North Indian elderly outpatients with dementia. Ann. Indian Acad. Neurol..

[63] Zhang M., Gong W., Zhang D., Ji M., Chen B., Chen B., Li X., Zhou Y., Dong C., Wen G. (2022). Ageing related thyroid deficiency increases brain-targeted transport of liver-derived ApoE4-laden exosomes leading to cognitive impairment. Cell Death Dis..

[64] Booms S., Hill E., Kulhanek L., Vredeveld J., Gregg B. (2016). Iodine Deficiency and Hypothyroidism from Voluntary Diet Restrictions in the US: Case Reports. Pediatrics.

[65] Nepal A.K., Suwal R., Gautam S., Shah G.S., Baral N., Andersson M., Zimmermann M.B. (2015). Subclinical Hypothyroidism and Elevated Thyroglobulin in Infants with Chronic Excess Iodine Intake. Thyroid.

[66] Benvenga S., Famà F., Perdichizzi L.G., Antonelli A., Brenta G., Vermiglio F., Moleti M. (2022). Fish and the Thyroid: A Janus Bifrons Relationship Caused by Pollutants and the Omega-3 Polyunsaturated Fatty Acids. Front. Endocrinol..

[67] Wu K., Zhou Y., Ke S., Huang J., Gao X., Li B., Lin X., Liu X., Liu X., Ma L. (2021). Lifestyle is associated with thyroid function in subclinical hypothyroidism: A cross-sectional study. BMC Endocr. Disord..

